# Copper Sulfide Based Heterojunctions as Photocatalysts for Dyes Photodegradation

**DOI:** 10.3389/fchem.2019.00694

**Published:** 2019-10-23

**Authors:** Luminita Isac, Cristina Cazan, Alexandru Enesca, Luminita Andronic

**Affiliations:** Product Design, Mechatronics and Environmental Department, Transilvania University of Brasov, Braşov, Romania

**Keywords:** copper sulfide, semiconductors, heterojunctions, nanostructures, dyes, photocatalysis

## Abstract

The presence of toxic, non-biodegradable and harmful organic pollutants in soils, wastewater, and atmosphere has become an indisputable, and global fact as a significant environmental problem. The heterogeneous photocatalysis, an advanced oxidation process (AOP) using semiconductor materials as catalysts, is a topic of great interest considering the possibility of the pollutants removal from water. The photocatalytic degradation of organic contaminants (i.e., dyes, pesticides, phenolic compounds) present in water using semiconductor materials depends on a number of parameters such as: the bandgap energy, phase composition, crystallinity, morphology and surface area of catalyst, electron-hole recombination rate, intensity of light, and adsorption capacity of the dye on the photocatalyst surface. One of the important constraints related to the catalyst photocatalytic efficiency is the fast recombination of the photogenerated electrons and holes. Therefore, various strategies have been involved in promoting the charge separation, including the development of heterojunction between two semiconductor materials, by tailoring the photocatalysts properties. This mini-review deals with the recent developments on dyes photodegradation using as catalysts various heterojunctions based on copper sulfide nanostructures, such as copper sulfide/metal oxide, copper sulfide/metal sulfide, copper sulfide/graphene, copper sulfide/organic semiconductors etc. The effects of different parameters, such as synthesis parameters, particle size, bandgap energy, surface area, and morphology on the photocatalytic activity of copper sulfide heterojunctions for dyes degradation is also highlighted.

## Introduction

Heterogeneous photocatalysis, an advanced oxidation process, requires energy and solar energy can be a viable source, for carefully selected catalysts, meaning materials with high absorption capacity for solar light, and conductivity for photogenerated charge carriers. Therefore, the design and development of semiconductor materials with wide solar selective spectral response, increased activity and stability are key targets in improving the efficient use of solar energy in attractive research topics: solar energy conversion and environmental treatment, such as air purification, water disinfection and purification, and hazardous waste remediation (Peng et al., 2009; Yang et al., 2019).

An important class of semiconductor-based photocatalysts is metal sulfides, for which the bandgap can be slightly tuned by simply controlling the particle sizes, without altering the chemical composition (Zhang et al., 2019).

Among metal sulfides, copper sulfides with different morphologies, and bandgap values are recognized as important semiconducting materials with potential applications in many fields such as solar cells (Isac et al., 2011; Yan et al., 2013), photocatalysis (Cao et al., 2015; Srinivas et al., 2015; Ayodhya et al., 2016; Li et al., 2017; Wu et al., 2017), lithium-ion rechargeable batteries (Yang et al., 2018), supercapacitors (Krishnamoorthy et al., 2014), electrochemical biosensing (Liu et al., 2014), photothermal conversion (Fang et al., 2018; Rokade et al., 2018), and as materials exhibiting localized surface plasmon resonance (LSPR) absorption (Jia et al., 2017).

Many of recent research focuses on designing and developing highly active, stable, and low-cost photocatalytic materials with enhanced solar energy conversion efficiency. Doping or surface modification can improve the absorption of Vis light, but photo-induced electron, and hole recombination still remain an unsolved problem in a single semiconductor. The most common way used to enhance the photocatalytic performance of semiconductors is the development of heterojunction structures with at least two advantages: broadening the photoresponse range of the semiconductor materials, and improving the efficiency of the separated photogenerated electrons from holes (Yang et al., 2019).

The recent developments related to the photocatalytic activity of various heterojunctions/composites based on copper sulfide nanostructures for organic dyes degradation under simulated irradiation (UV, Vis and different combination between both) and sunlight irradiation were extensively discussed in this mini-review.

## Dyes Photodegradation—a General Mechanism Overview

Most dyes are synthetic, including cationic, anionic (acid, reactive, and direct dyes) and non-ionic dyes. Related to chemical structure, they are classified in azo, anthraquinone, indigoid, nitroso, and nitro dyes. Dyes are frequently used in many industries (textile, leather, paper, rubber, printing, plastics) and are chemically stable and resistant to degradation, thus remaining in water for a long time. A large number of dyes discharged in water causes a huge risk to the environment because dyes can reduce sunlight transmission and contain toxic substances, such as heavy metals (lead, chromium) and aromatic compounds (Pavithra et al., 2019).

Dyes are the most model of pollutant used for evaluation of the photocatalytic activity of the catalyst. Researchers have studied the light assisted photodegradation of many organic dyes, such as azo dye (methyl orange—MO, rhodamine B—RhB, congo red—CR, acid orange 7—AO7), thiazine, and cationic dyes (methylene blue—MB) (Natarajan et al., 2018).

**Figure 1:**
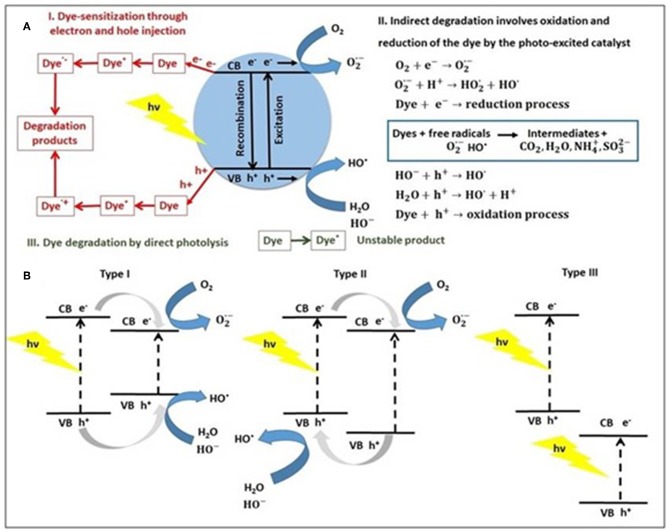
**(A)** The general photocatalytic degradation mechanisms of dye using a single semiconductor (e.g., metal sulfide) and **(B)** type of semiconductor heterojunctions based on metal sulfide, under light irradiation.

The photocatalytic degradation mechanisms of dyes using a photocatalyst are summarized in Figure 1A as (I) dye-sensitization through charge injection, (II) indirect dye degradation through oxidation/reduction, and (III) direct photolysis of dye (Pingmuang et al., 2017).

In type I mechanism, the charge carriers (electron-hole pairs) generated in the presence of light can interact with dye molecules (Dye) through the chemisorption mechanism to produce excited dye (Dye^*^) that can be converted to cationic (Dye^·+^) and anionic (Dye^·−^) radicals, which leads to degradation products (Natarajan et al., 2018). Dye sensitization is an efficient method to modify photocatalyst due to some crucial reasons: expands the light absorption to visible light irradiation making use of solar energy, enhances the excitation efficiency and also promote the electron-hole separation, thus leading to higher photocatalytic performance.

In type II mechanism, when the semiconductor photocatalyst absorbs photons from solar light or irradiation source, with energy equal, or higher than its band-gap, an electron (e^−^) from the valence band (VB) is promoted to the conduction band (CB) is occurred. This is associated with a hole (h^+^) generation in the valence band, to form pairs of electrons and holes, participating at reduction, and oxidation reactions (Al-Mamun et al., 2019). The electron-hole recombination on the surface represents the mechanism that deactivates the photocatalyst. Also, electrons and holes trapping in surface states lead to reactions with molecular oxygen (O_2_) to produce anionic superoxide radicals ($O_{2}^{\cdot -}$) and photogenerated holes react with water forming hydroxyl radicals (HO^·^), which further oxidize the dye molecules. The photogenerated electrons and holes can generate the most essential hydroxyl radical (HO^·^) well-known as a highly reactive radical (Ng et al., 2019). The hydroxyl radicals act as reactants for dyes oxidation in wastewater photocatalysis, to transform pollutants into CO_2_ and H_2_O. When dyes molecules contain heteroatoms (e.g., sulfur, nitrogen, halogens, phosphorus), they are converted into corresponding salts ions, environmental-friendly compounds that do not require any other chemical or physical treatment (Chun et al., 2013). The direct photolysis of dye molecules (mechanism type III) is a slow process and is independent of the catalyst.

To improve the performance of semiconductors in photocatalysis, many heterojunctions are developed in the last years. A heterojunction is defined as an interface between two semiconductors with the unequal band structure, according to the different charge carriers separation mechanisms. Therefore, three types of sulfide-based heterojunction photocatalysts have been reported (Bu, 2014; Ren et al., 2019) to date: type I—straddling gap, type II—staggered gap and type III—broken gap. By exposure to light, both heterojunction semiconductors could be excited to produce photogenerated electrons (e^−^) and holes (h^+^). In the Type I heterojunction, the band energy levels of one semiconductor are included in that of the second one, and the electrons and holes migrate along the same direction (Zhang et al., 2019). In the opposite case (type II heterojunction), the electrons and holes are allowed to transfer to other semiconductors from heterojunction, when spatial charge separation has occurred. The general mechanism of dyes using a heterojunction photocatalyst was explained above and is embodied in the forthcoming chapter.

## Photodegradation of Dyes Using Heterojunctions Based on Copper Sulfide Nanostructures

Many heterojunction based on copper sulfide nanostructures were developed in our group in last 10 years, the one reason of the extensive research was to extend the photocatalytic response of the materials in VIS region of solar spectrum, for industrial applications, thus a significant decrees in the operation costs. Thin films tandem structures, such as Cu_x_S/SnO_2_, ZnO/Cu_x_S-CuO/SnO_2_, and TiO_2_/Cu_x_S-CuO/SnO_2_, obtained by the successive deposition of individual semiconductor layers using Spray Pyrolysis Deposition (SPD), allow high charge carriers mobility, able to generate oxidative species during photocatalysis. The Cu_x_S/SnO_2_ and ZnO/Cu_x_S-CuO/SnO_2_ were tested by Enesca et al. (2015) using 0.0125 mM methylene blue as a pollutant and mixt of UV (3 lx flux intensity) and Vis radiation (28 lx flux intensity). The results after 6 h of irradiation indicate that the ZnO/Cu_x_S-CuO/SnO_2_ tandem system have a higher contribution of cumulative charge generation allowing to reach 78% photocatalytic efficiency, while Cu_x_S/SnO_2_ attempt 74% photocatalytic efficiency. The contribution of each component on the overall photocatalytic efficiency is based on the suitable disposal of the energy levels. The tandem system based on TiO_2_/Cu_x_S-CuO/SnO_2_ was tested by Duta et al. (2016) using the same irradiation scenario but exchanging the pollutant. In this case, the pollutant was phenol in different concentration of 4, 10 and 20 ppm, after 6 h of irradiation the highest efficiency was 8.75% for the 4 ppm pollutant concentration.

The photocatalytic process using heterojunctions Cu_x_S/TiO_2_ tandem semiconductors in the dyes (MB and MO) photodegradation was also investigated in our group (Andronic et al., 2011), which pointed out the improved photocatatytic activity of heterojunction based nanostructured materials in dyes photodegradation, compared to single-component nanomaterials, or one-phase heterojunctions. Copper sulfide powder, containing predominant crystalline phases CuS and Cu_1.8_S (digenite), was synthesized via the photochemical method and thin films of Cu_x_S, TiO_2_ and coupled Cu_x_S/TiO_2_ was deposited on the glass by doctor blade technique. The higher photocatalytic activity of Cu_x_S/TiO_2_ heterojunction structure is Type II (Figure 1), is explained by the irreversible charge separation, which causes kinetic constraints as result with dye pollutant interactions. The photocatalytic activity of Cu_x_S/TiO_2_ photocatalysts depends on the molar ratio of Cu_x_S:TiO_2_, the best results corresponding to Cu_x_S:TiO_2_ = 3:7 when high degradation efficiency (almost 99%) was obtained after 180 min for MB degradation, and after 300 min for MO degradation, respectively.

Many copper sulfide-based heterojunctions, classified as copper sulfide/metal oxide, copper sulfide/metal sulfide, and copper sulfide/carbon-based materials heterojunctions developed by scientific community were summarized in Table 1.

**Table 1:** Representative studies on dyes photocatalytic degradation using various heterojunctions based on nanostructured copper sulfides as photocatalysts.

**Photocatalyst/catalyst dosage**	**Synthesis method**	**Dye (conc.)**	**Light type**	η%^*^	*t* (min)^**^	**References**
**COPPER SULFIDE/METAL OXIDE HETEROJUNCTION PHOTOCATALYSTS**						
CuS/TiO_2_ 3.33 g/L	CuS loaded on rutile TiO_2_	Orange II (20 mg/L)	Vis	60	90	Lu et al., 2016
CuS/TiO_2_ 0.5 g/L	CuS sonochemical precipitation on TiO_2_ nanofibers	MB (5·10 ^−6^ M)	Vis	86	180	An et al., 2018
Cu_x_S/TiO_2_ 1 g/L	*In situ* synthesis	MB (10 mg/L)	Vis	95	180	Gao et al., 2017
CuS/CuO 1 g/L	Thermal oxidation	MB (10 mg/L)	Vis	89	240	Kao et al., 2019
Cu_7_S_4_/Cu_2_O 1 g/L	Chemical etching reaction with CuO	MO (10 mg/L)	Vis	37	100	Li et al., 2016
Cu_2_S/T-ZnO_w_ 1 g/L	Simple polyol process	MO (10 mg/L)	Vis	97	120	Wu et al., 2012
CuS/ZnO 0.4 g/L	Wet-chemical	MB (2·10^−5^ M)	Vis	87	30	Basu et al., 2014
**COPPER SULFIDE/METAL SULFIDE HETEROJUNCTION PHOTOCATALYSTS**						
CuS/MoS_2_ 0.3 g/L	Hydrothermal	MB (10 ppm)	Solar	100	60	Meng et al., 2015
CuS/CdS 1 g/L	Hydrothermal	MB + H_2_O_2_ (10 ppm)	Vis	99.97	10	Mahanthappa et al., 2019
CuS/ZnS 1 g/L	Hydrothermal	RhB (1·10^−5^ M)	Vis	50	120	Thuy et al., 2014
CuS/CdS/TiO_2_ 1 g/L	Hydrothermal	AO7 (5 mg/L)	Vis	100	120	Maleki and Haghighi, 2016
ZnO/ZnS/CuS 1 g/L	Three-step chemical route	MO (10 mg/L)	Vis	98	210	Liu et al., 2014
**COPPER SULFIDE/CARBON-BASED MATERIALS HETEROJUNCTIONS PHOTOCATALYSTS**						
CuS/Gr 0.4 g/L	Hydrothermal	MB+H_2_O_2_ (80 mg/L)	UV+Vis	93	80	Wang et al., 2014
CuS/rGO 2 g/L	Solvothermal	MB (15 mg/L)	Vis	80	140	Hu et al., 2017
CuS/GO/TiO_2_ 1 g/L	Sol-gel	MB (50 mg/L)	Vis	90	120	Park et al., 2013
CuS/g-C_3_N_4_ 1 g/L	Hydrothermal	MB (10 ppm)	Vis	96	90	Khan et al., 2018
CuS/g-C_3_N_4_ 0.3 g/L	*In-situ* synthesis	MB (10 mg/L) RhB (10 mg/L)	Vis	85–98 96.8	120	Cai et al., 2017

^*^η is the efficiency degradation of dyes after t^**^ min of irradiation.

Heterojunctions of CuS/TiO_2_ were obtained via an environmentally friendly route that uses biomolecule L-cysteine as a sulfur source and chelating agent for *in situ* synthesis of CuS nanoparticles on the surface of 1D TiO_2_ nanobelts, thus solving the problem related to the aggregation of CuS nanoparticles (Gao et al., 2017). The degradation of MB was used to evaluate the photocatalytic activity of the CuS/TiO_2_ samples under Vis light (350 W Xe lamp with a UV cutoff filter, λ ≥ 420 nm) irradiation. The results indicated that degradation efficiencies of CuS/TiO_2_ heterojunction based photocatalysts, in the range 95–100%, are more than twice of that of pure TiO_2_ nanobelts (46%). The enhanced photocatalytic activity was attributed to the energy bandgaps match between CuS and TiO_2_, resulting in efficient separation of photogenerated electrons and holes.

Recent research (Kao et al., 2019) was focused on the photocatalytic properties of CuO/CuS core-shell nanowires, prepared through thermal oxidation and two-step annealing, used for the degradation of methylene blue under Vis light irradiation. The photocatalytic tests pointed out that the MB degradation rate after 4 h was increased by almost 25% for the CuO/CuS heterojunction compared to CuO nanowires.

The enhanced visible-light photocatalytic efficiency of type II heterojunction ZnO/CuS in MB degradation was also reported (Basu et al., 2014). The ZnO/CuS photocatalyst was obtained by decorating CuS nanostructure on the surface of ZnO nanotubes, using a wet-chemical method at low temperature. The photocatalytic experiments showed that ZnO has no photocatalytic activity under Vis light irradiation, the MB photodegradation rate with CuS reaches 63% after 30 min, and with the ZnO/CuS catalyst, the photocatalytic efficiency increased to 87%, meaning almost 28% increase. According to the mechanism study, the enhanced photocatalytic activity is attributed to the ZnO/CuS p-n heterojunction formation, which favors the efficient separation of photoinduced charge carriers.

Using simple and eco-friendly hydrothermal method, CuS/CdS and CuS/MoS_2_ heterojunctions were synthesized (Meng et al., 2015; Mahanthappa et al., 2019). Based on the photocatalytic results, as compared to pure CuS, CdS, and MoS_2_, the CuS/CdS and CuS/MoS_2_ heterostructures showed higher photocatalytic performance toward the degradation of MB dye under Vis light (250 W W lamp with illumination intensity of 0.01 W/cm^2^), respectively simulated natural light (Xe lamp, 300 W) irradiation. It has been reported that CuS/CdS catalyst degraded 99.97% of MB (10 ppm), in presence of H_2_O_2_, after 10 min of exposure in Vis light. The CuS/MoS_2_ catalyst degraded MB solution (10 ppm) after 60 min exposure to sunlight. The enhanced photocatalytic degradation efficiencies of CuS/CdS and CuS/MoS_2_ photocatalysts are attributed to the significant reduction in the recombination of electron-hole pairs.

Recently, CuS/graphene (graphene oxide) composites are recognized as very efficient photocatalysts for dyes degradation, due to the graphene (GR)/graphene oxide (GO) specific properties: large surface area, porous morphology, the ability to photogenerated electrons across the composite interface, and strong adsorption capacity for organic molecules. In this context, three CuS/graphene-based photocatalysts were prepared and used for the evaluation of MB photodegradation under Vis light irradiation: CuS nanocrystals/GR nanocomposites with different GR weight ratios (1, 5, 10, 20%) obtained by hydrothermal method (Wang et al., 2014), flower-like CuS/rGO composites synthesized by a facile one-step solvothermal procedure (Hu et al., 2017), and CuS-GO/TiO_2_ composites prepared via a sol-gel method (Park et al., 2013). In CuS/GR composites, CuS nanocrystals with average diameter of 16.2 nm are uniformly and completely dispersed on GR nanosheets, like bees lying on honeycombs. This porous morphology is suitable for enhancing light absorption and adsorption of dye molecules. The ability of CuS/GR composites to degrade MB solution, in presence of H_2_O_2_, under UV and Vis light irradiation was investigated. The results showed that the MB degradation efficiency was about 30% higher for CuS/GR(10%) catalyst than that for CuS nanocrystals, after 80 min of irradiation with UV/Vis light, which means a significant increase. The high stability and photoactivity of CuS/GR were assigned to the high electronic conductivity of graphene and its significant influence on the morphology of the CuS/Gr nanocomposite (Wang et al., 2014).

The photodegradation of MB solutions, without the assistance of H_2_O_2_ which obviously accelerate the dye degradation, was carried out to evaluate the photocatalytic activity of CuS/GO and CuS/rGO (reduced graphene oxide) based catalysts under Vis light irradiation. If in CuS/rGO composites catalysts, CuS microflowers with average diameter around 2–8 μm are relatively uniformly distributed and decorated on the rGO sheets, in CuS-GO/TiO_2_ catalysts, fine TiO_2_ particles are agglomerated randomly on the GO surface. Generally, high photocatalytic activity is associated with uniform particles dispersion on rGO/GO sheets, what is confirmed for CuS/rGO catalyst, and/or porous morphology, attributed to CuS-GO/TiO2 catalyst due to the pores developed on the GO surface. The specific surface area of a photocatalyst together with micro- mesopores size distribution have a significant effect on its photocatalytic activity. The rGO sheets, with high BET surface (34.8 m^2^/g) enhance the specific surface area of CuS/rGO composite by favoring the uniform deposition of more CuS microflowers on rGO surface. As a result, calculated BET surface area was 10.6 m^2^/g, almost twice as large as that of pure CuS (5.8 m^2^/g). Compared to GO, with very large specific surface of 1,083 m^2^/g, the BET surface area drastically decreased to about 3.57 and 13.29 m^2^/g for the CuS-GO and CuS-GO/TiO_2_ composites, respectively. This decrease was associated with the significant change in micropore size distribution for the CuS-GO/TiO_2_ composites compared with that of GO. The photocatalytic efficiency of CuS/rGO catalyst in MB degradation under Vis light irradiation for 140 min, was increased to nearly 80%, that means about 4 times, respectively 2 times higher than that of pure CuS and rGO, respectively. After 120 min under Vis light irradiation, the MB degradation efficiency with CuS-GO/TiO_2_ catalyst was approximately 33%, 2.3 times higher than that with CuS-GO composite. The enhanced MB degradation efficiency for CuS/GO based photocatalysts was attributed to the effect of GO in CuS-GO/TiO_2_, respectively, rGO in CuS/rGO heterojunction structure, by increasing both the charge mobility of excited photoelectrons to the conduction band of TiO_2_/CuS, promoting the efficient charge carriers separation, and specific surface area (more reactive sites). As conclusion, the GO/rGO has important contributions to MB photodegradation with CuS/GO (rGO) hybrid photocatalysts, such as: (a) ensures relative large specific surface area which provides more active reaction sites exhibiting strong photoabsorption of MB molecules under Vis light; (b) favors meso- or microporous surface morphologies enabling the migration of MB molecules in the surface pores; (c) allows strong adsorption of MB molecules due to the oxygen-containing functional groups existing on the surface of GO/rGO. All these contributions have as result significantly improved photocatalytic activity of these types of photocatalysts due to an integrative combination of GO/rGO and CuS/TiO_2_ particles.

Organic semiconductors, such as graphitic carbon nitride (g-C_3_N_4_) could also act as a component of a heterojunction based photocatalyst. Several studies (see Table 1) were focused on the photocatalytic performances of the heterojunction CuS/g-C_3_N_4_, compared with single CuS and g-C_3_N_4_ components, in dyes (MB, RhB) degradation under Vis light irradiation.

The obtaining of the CuS/g-C_3_N_4_ heterojunctions by a precipitation-deposition method at low temperature, using different amount of porous g-C_3_N_4_ (100, 200, and 300 mg), was also reported (Chen et al., 2016). The photocatalytic activity of CuS/g-C_3_N_4_ composites was evaluated for the decomposition of various dyes (RhB, MB) aqueous solutions in Vis light (300 W Xe lamp, λ ≥ 420 nm) irradiation. In the degradation of MB, the calculated rate constant was 3.57% min^−1^, when CuS/g-C_3_N_4_ is used as photocatalyst, value which is 21 times or 10.2 times higher than those for g-C_3_N_4_ or CuS. Related to the RhB photodegradation, all the CuS/g-C_3_N_4_ samples showed enhanced photocatalytic activities compared with g-C_3_N_4_ (5% degradation efficiency) and CuS (40% degradation efficiency), the highest photodegradation efficiency, after 60 min irradiation in Vis light, was about 99.6%. According with this study, the enhanced photocatalytic activity of CuS/g-C_3_N_4_ heterojunction could be attributed to the matching of the g-C_3_N_4_ and CuS bandgap energies.

As well, high photocatalytic activity and stability in the degradation of RhB dye aqueous solutions under Vis light (400 W metal halide lamp) irradiation showed the type II heterojunction CuS/CCN nanostructures (Figure 1). Nanoflowers (NFs) CuS/CCN (carbon self-doped g-C_3_N_4_) materials were prepared via a mildly hydrothermal method, using different amounts (1, 2, 5, 10 wt.%) of CCN (Wang et al., 2016). The variation of CCN content in CuS/CCN samples has a significant influence on the CuS nanoparticles morphology and consequently on the CuS/CCN morphology and photocatalytic properties. According with photocatalysis results, the CuS NFs/CCN (5% wt) could degrade about 92.6% RhB solution in 180 min, exhibiting a degradation rate of 2.8 times higher than that of CuS NFs. It is obviously that the formation of p–n heterojunctions, with n-type CCN and p-type CuS semiconductors, not only reduce the charge transfer resistance but contributes to the efficient separation of photogenerated electron–hole pairs.

The photocatalytic degradation of carcinogenic Congo Red (CR) dye, under sunlight irradiation, with CuS/rGO semiconductor based nanocomposites, was the subject of a research performed by Borthakur's group (Borthakur et al., 2016). The CuS/rGO heterostructure was synthesized using a simple and green microwave irradiation technique. The photocatalytic experiments of CR dye degradation were carried out in presence of CuS/rGO at different initial dye concentration (100, 150, 200, 250, 300 mg/L) and in the pH range 3–11. Considering the optimized working conditions (catalyst concentration 300 mg/L; dye concentration: 100 mg/L; suspension pH = 5), the reaction mixture was first kept in dark for 2 h, to measure the adsorption/desorption equilibrium, and then was exposed to sunlight for 1 h. It was demonstrated that the presence of rGO in CuS/rGO nanocoposite induces a synergistic effect between CuS and rGO sheets, which leads to higher efficiency of CR dye photocatalytic degradation compared to CuS nanoparticles (no rGO support) and single rGO.

The malachite green (MG) aqueous solutions, with concentrations of 5·10^−6^ mol/L and pH 7, were used to study the photocatalytic activity of Fe-doped TiO_2_ nanotubes (NTs) and CuS/Fe-doped TiO_2_ hybrid material under sunlight irradiation with average intensity of ~900 W/m^2^ (He, 2016). The sunlight-excited degradation of MG in the water showed higher photocatalytic activity for hybrid CuS/Fe-doped TiO_2_ nanostructures, compared with that of NTs, and increased with increasing CuS/NTs ratio. The enhanced photodegradation of MG dye (~ 90% after 150 min) in presence of CuS/Fe-doped TiO_2_ (Eg = 2.50–2.85 eV) was attributed to the effects of the light-generated electrons and holes, larger BET surface area (~794–1,100 m^2^/g), and heterojunction between the NTs (Eg = 2.95 eV) and CuS nanoparticles (Eg = 3 eV).

Heterostructured ZnS-ZnO-CuS-CdS photocatalyst was prepared using a sequential fabrication approach (ZnS NPs → thermal treatment → ZnS-ZnO composite → CuS formation → tri-component ZnS-ZnO-CuS heterostructure → CdS addition → four-component ZnS-ZnO-CuS-CdS heterostructure). It is interesting that, in this four-semiconductor structure, each component has its own role in the photocatalytic degradation of methyl blue (MeB) under 1 sun irradiation (100 mW/cm^2^, using AM 1.5 G filter). To improve photocatalytic degradation of MeB dye, after the optimization of oxide content in the ZnS-ZnO composites, controlled by thermal processing conditions, the metal sulfides (CdS and CuS) were added as light sensitizer and co-catalyst, respectively (Hong et al., 2015).

In recent years, many researches highlighted the extensive potential applications of the metal oxide/copper sulfide-based materials with improved physical, chemical, and photocatalytic properties. Therefore, the photocatalytic decolorization of anionic dye methyl orange (MO) aqueous solution, at ambient temperature under Vis light (500 W Xe lamp) irradiation, using Cu_2_O/Cu_7_S_4_ heterojunction catalyst was studied (Li et al., 2016). The uniform and monodispersed Cu_2_O/Cu_7_S_4_ crystals with core-shell structure, synthesized by a simple chemical etching reaction, showed enhanced photocatalytic activity compared to Cu_2_O crystals. Maintaining the dispersion pH at 7, after 100 min in Vis light irradiation, 11.27% of MO was degraded by Cu_2_O crystals, while about 90% of MO was degraded by the Cu_2_O/Cu_7_S_4_ catalyst, i.e., almost eight times more. This performance is associated with the unique Cu_2_O/Cu_7_S_4_ core-shell structure consisting of small Cu_7_S_4_ particles easily aggregated on the surface of cuboctahedron Cu_2_O microcrystals with an average particle size of 1.1 μm. In Cu_2_O/Cu_7_S_4_ heterostructure, the small Cu_7_S_4_ particles can act as a sink for the electrons, thus the interfacial charge-transfer between the two semiconductors is promoted and the recombination of free electrons and holes is prevented. In addition, on the surface of Cu_2_O microcrystals, pores of 9 nm size (larger compared with 4 nm pore size in Cu_2_O) are formed between Cu_7_S_4_ particles, supporting the good absorption capacity of catalyst, while its improved adsorptive ability in the photocatalytic process is due to the interstice developed between the inner Cu_2_O core and Cu_7_S_4_ shell.

Investigations were made to evaluate and compare the MO aqueous solution (100 mL, 1·10^−5^ mol/L) photodegradation using pristine/Graphene (Gr), Graphene/ZnO (GZ), and Graphene/CuS/ZnO (GCZ) hybrid composites under UV light irradiations (Varghese and Varghese, 2015). The photodegradation efficiency of MO within 30 min was 42, 64, and 99% for Gr, GZ and GCZ photocatalysts. The enhanced photocatalytic activity of GCZ hybrid catalyst is mainly due to its high surface area (635 m^2^/g) resulting in a stronger electrostatic attraction between MO molecules and GCZ particles.

The lack of standards in the field of photocatalysis and photocatalytic heterojunctions make it impossible to compare the results provide by different researchers, with all these inconveniences, some indicative optimized parameters can be given: the catalyst concentration between 0.5 and 2 g/L, the dyes concentration around 10 mg/L.

## Conclusions

Semiconductor(s) based photocatalysts are recognized as promising materials for industrial applications, combining the advantage of low cost with the fulfillment of the appropriate requirements in order to increase the efficiency of the photocatalytic process.

Generally, metal oxides with wide bandgaps (e.g., ZnO, SnO_2_, TiO_2_) are photoactive in UV light radiation domain, thus limiting their efficiency, while most of the metal sulfides (e.g., CdS, CuS) show better absorption in Vis region but are prone to photo-corrosion and have relatively low chemical stability. To improve the photocatalytic activity in VIS range of metal oxides, copper sulfide-based heterostructures were prepared by facile, low-cost, and energy-efficient methods: hydrothermal/solvothermal methods, sol-gel processes, *in-situ* synthesis, microwave irradiation, chemical precipitation etc., easy to prepare at industrial level to transfer these materials in industrial applications. A higher photocatalytic performance (between 80 and 100% of dyes degradation) of various heterojunctions based on copper sulfide nanostructures for dyes photodegradation pointed out in this mini-review demonstrate that these materials may act as VIS active materials, stable and low-cost photocatalysts with enhanced solar energy conversion efficiency for environmental remediation and green energy production.

## Author Contributions

LI planned the contents and wrote the draft. CC contributed to the article database. AE advised and supervised the work of the team. LA initiated and actively contributed to manuscript writing.

### Conflict of Interest

The authors declare that the research was conducted in the absence of any commercial or financial relationships that could be construed as a potential conflict of interest.
